# Angiotensin I Converting Enzyme Inhibitory Peptides Obtained after *In Vitro* Hydrolysis of Pea (*Pisum sativum* var. Bajka) Globulins

**DOI:** 10.1155/2014/438459

**Published:** 2014-08-28

**Authors:** Anna Jakubczyk, Barbara Baraniak

**Affiliations:** Department of Biochemistry and Food Chemistry, University of Life Sciences, Ulica Skromna 8, 20-704 Lublin, Poland

## Abstract

Pea seeds represent a valuable source of active compounds that may positively influence health. In this study, the pea globulins were digested *in vitro* under gastrointestinal condition and potentially bioaccessible angiotensin I converting enzyme (ACE) inhibitory peptides were identified. The degree of hydrolysis after pepsin, 14.42%, and pancreatin, 30.65%, were noted. The peptides with the highest ACE inhibitory properties were separated using ion exchange chromatography on DEAE-cellulose. Thirteen peptides fractions were obtained but only four showed potential antihypertensive properties. The highest inhibitory activity was determined for the fraction F8 (IC_50_ = 0.0014 mg/mL). This fraction was separated on Sephadex G10 and two peptide fractions were obtained. The peptides fraction (B) with the highest ACE inhibitory activity (IC_50_ = 0.073 mg/mL) was identified by ESI-MS/MS. The sequences of ACE inhibitory peptides were GGSGNY, DLKLP, GSSDNR, MRDLK, and HNTPSR. Based on Lineweaver-Burk plots for the fraction B, the kinetic parameters as *K*_*m*_, V_max_, and *K*_*i*_ and mode of inhibition were determined. This fraction belongs to uncompetitive inhibitor of ACE activity. The seeds of pea are the source of precursor protein, which releases the ACE inhibitory peptides as a result of enzymatic hydrolysis.

## 1. Introduction

In recent years, food has become the subject of research not only as a source of energy and basic nutrients, which are essential for proper development and functioning of the body, but also as a source of bioactive compounds that may have the positive influence on health condition [1–3]. The proteins are very important component of diet providing necessary amino acids for the proper functioning of the body and may be precursors of active peptides, which as a result of food processing or release during gastrointestinal digestion have physiological activity [4].

One of the most important parts of human homeostasis is the renin-angiotensin-aldosterone system (RAS) whose influence on the regulation and functioning of the circulatory system and water-electrolyte has been known for many years. This system is associated with the maintenance of normal blood volume, blood pressure stability, and proper amount of sodium ions [5]. Disorder in its functioning is the basis for the pathogenesis of diseases such as heart failure, coronary artery disease, hypertension, diabetes complications, or kidney disease [6, 7]. Angiotensin converting enzyme (ACE, EC 3.4.15.1), zinc metalloprotease, plays the most important role in this system and converts the inactive angiotensin I (NRVYIHPFHL) into the potent vasoconstricting angiotensin II (NRVYIHPF) and the vasodilator bradykinin into an inactive peptide leading to an increase in blood pressure [8, 9]. The beneficial effect of inhibitors of ACE is the reduction of angiotensin II in plasma, as well as increased levels of bradykinin. This results in preventing the contraction of the heart and blood vessels, positive effect on endothelial function, and an anti-arteriosclerosis and regulation of blood clotting mechanism [10]. Nowadays, many synthetic drugs (such as captopril, quinapril, enalapril, or ramipril) whose action is based on the inhibition of ACE activity which affects the proper functioning of the body and protects the heart muscle are used. Unfortunately, they may cause serious side effects and therefore the search of alternative inhibitory of this enzyme has increased. One of them can be peptides-natural compounds in the free state as secondary metabolites or released during the enzymatic hydrolysis of plant or animal proteins [11]. Many ACE inhibitory peptides have been purified and identified from plant foods protein such as protein from some legume species, cowpea, soybean, chickpea, and pea [12–14], or other plants and products, fermented pea [15], cottonseed [16], or garlic [17]. Legume seeds are becoming increasingly frequent part of the diet due to not only the taste qualities but also nutrition.

The aim of this study was the identification of potentially bioaccessible ACE inhibitory peptides obtained by digestion under simulated gastrointestinal conditions of pea globulins. Pea seeds are a valuable source of protein, minerals, carbohydrates, vitamins, or active compounds: peptides or antioxidants. To data, no study has conducted the investigation of primary structure of angiotensin converting enzyme inhibitors from pea globulins after* in vitro* digestion.

## 2. Materials and Methods

### 2.1. Materials

Pea seeds (*Pisum sativum* var. Bajka) were obtained from Company of Horticulture Seeds and Nursery in Ożarów Mazowiecki, Poland. HHL (hippuryl-L-histidyl-L-leucine), pepstatin A, PMSF (phenylmethanesulfonyl fluoride), α-amylase from hog pancreas (50 U/mg, 10080, Sigma), pepsin from porcine gastric mucosa (250 U/mg, P7000, Sigma), pancreatin from porcine pancreas (P7545, Sigma), enalapril, bile extract, and TNBS (2,4,6-trinitrobenzenesulfonic acid) were purchased from Sigma-Aldrich Company, USA, and Amino Acid Standard from Pierce, USA. Any other chemicals were of analytical grade.

### 2.2. Preparation of ACE from Pig Lung

Angiotensin converting enzyme was prepared according to the procedure of Hayakari et al. [18] with slight modifications. Pig lungs were purchased in a local market and used as a starting material. Lung tissues (100 g) were homogenized in 0.1 M borate buffer pH 8.3 containing pepstatin A (0.1 mM) and PMSF (0.1 mM) at 4°C in ratio 1 : 2 (w/v). The homogenate was centrifuged at 8000 ×g, 4°C, for 20 min. The purification of ACE was initiated by the addition of solid ammonium sulphate at 80% saturation and next dialyzed (molecular weight cut-off 12 kDa) for 24 h at 4°C against 20 volume of 0.1 M borate buffer pH 8.3. The dialysate sample was centrifuged at 8000 ×g, 4°C, for 20 min. The ACE activity of the dialysate was assayed with the method below, frozen, and used for further analysis.

### 2.3. Isolation of Globulins

Globulins were isolated from pea flour according to Gupta and Dhillon [19] with modification: endoproteases and their inhibitors were inactivated by heating in laboratory oven at 100°C for 10 min to inactivate endoproteases and their inhibitors. Flour was dispersed in 0.2% NaOH in ratio 1 : 10 (w/v) and protein extraction was carried out under stirring for 1 h at room temperature. The solution was centrifuged for 20 min at 8000 ×g, 4°C. After that, pH of supernatant was adjusted to the isoelectric point of pea globulins (pH 4.3) with 0.1 M HCl. Precipitated globulins were centrifuged for 20 min, 4°C, 8000 ×g and were washed with distilled water. Globulins were stored at −18°C until further use.

### 2.4. *In Vitro* Digestion and Absorption

*In vitro* digestion of pea globulins was carried out according to the method described by Gawlik-Dziki [20] with slight modification. Briefly, the lentil globulins (4%, w/v) were resuspended in stimulate saliva solution with final concentration 7 mM NaHCO_3_ and 0.35 mM NaCl, pH 6.75, and stirred for 5 min at 37°C in darkness. After that, α-amylase (50 U/mg) was added (the ratio of enzyme to substrate was 1 : 10; w/w) and the mixture was stirred for 10 min at 37°C in darkness. For the gastric digestion, solution was adjusted to pH 2.5 with 1 M HCl and pepsin (250 U/mg) was added (the ratio of enzyme to substrate was 1 : 100; w/w). Reaction was carried out for 2 h at 37°C. Solution was neutralized to pH 7.0 with 1 M NaOH and successively a mixture containing 0.7% solution of pancreatin and 2.5% solution of bile extract (1 : 2.5, v/v) was added (simulated intestinal digestion). The incubation was carried out for 1 h at 37°C in darkness and reaction was stopped by heating at 100°C for 5 min.

Hydrolysates were dialyzed with the dialysis sacks (D9777-100FT, molecular weight cut-off 12000, Sigma-Aldrich) against phosphate buffered saline (PBS) at the physiological concentration 1 : 4, v/v (simulated absorption process). The process was carried out in darkness for 1 h at 37°C. After this stage, the samples were concentrated tenfold using a vacuum evaporator; the parameters were set as follows: 40°C, pressure 0.8 mPa.

### 2.5. Determination of Free Amino Groups Content

Free amino groups content was determined by the trinitrobenzenesulfonic acid (TNBS) method using L-leucine as the standard [21]. All assays were performed in triplicate.

### 2.6. Determination of Degree of Hydrolysis (DH)

The degree of hydrolysis (DH) after* in vitro* digestion was estimated by determination of free amino groups by reaction with TNBS according to the method by Adler-Nissen [21]. The total number of all amino groups was determined in a sample by complete hydrolysis with 6 M HCl at 120°C for 24 h. All assays were performed in triplicate and the degree of hydrolysis was calculated from the formula

(1) $$\begin{array}{c} \text{DH}=\frac{h}{h_{\text{tot}}}\times 100\%, \end{array}$$

where DH is degree of hydrolysis, *h* are peptide bounds in the sample, *h*_tot_ are the total peptide bounds.

### 2.7. Assay for ACE Inhibitory Activity

ACE inhibitory activity was measured with 5 mM HHL as a substrate by spectrophotometric method according to Jakubczyk et al. [15]. Briefly, 50 *μ*L of peptide sample was added to 50 *μ*L of 5 mM HHL solution and 50 *μ*L of 3 mU/mL ACE (one unit of ACE activity was defined as an increase absorbance of 0.001 per minute at 390 nm). The reaction mixture was incubated at 37°C for 60 min. The reaction was terminated by adding 0.7 mL of the 0.1 M borate buffer with 0.2 M NaOH. The absorbance was measured at 390 nm and the ACE inhibition was determined as follows:

(2) $$\begin{array}{c} \text{ACE}\;\;\text{inhibition}\;\;\left(\%\right)=\left[1-\left(\frac{A1-A2}{A3}\right)\right]*100\%, \end{array}$$

where *A*1 is the absorbance of sample with ACE and peptide inhibitor, *A*2 is the absorbance of sample with peptide inhibitor, without ACE, and *A*3 is absorbance of sample with ACE and without peptide inhibitor but with 0.1 M borate buffer pH 8.3.

The IC_50_ (inhibitory concentration) was determined by assessing the ACE inhibition of several dilutions of each hydrolysate sample and interpolating the peptides concentration at which the inhibition percentage reached 50%. The IC_50_ value was calculated from the graph plotting inhibition for the five different peptide concentrations. Enalapril, applied in hypertension and chronic failure treatment as inhibitory of ACE, was used as the positive control.

### 2.8. Purification of ACE Inhibitory Peptide

#### 2.8.1. Ion Exchange Chromatography

The sample obtained after absorption process was separated using ion exchange chromatography on DEAE-cellulose (the column 1.5 × 14 cm). The concentration of peptides for ion exchange chromatography was calculated by soluble peptide content by the trinitrobenzenesulfonic acid (TNBS) method using L-leucine as the standard [21]. 2 mL of solution (3.19 mg/mL) was loaded on the column previously equilibrated with 0.1 M borate buffer pH 8.3 and was eluted with linear gradient of NaCl in 0.1 M borate buffer pH = 8.3 (0–0.8 M NaCl) at a flow rate of 0.8 mL/min. Two milliliters fractions were collected and the absorbance was monitored at 220 nm. Fractions with the highest absorbance were combined and concentrated on the vacuum evaporator (40°C, 0.8 mPa) and ACE inhibitory activity was determined. Fraction with the highest ACE inhibitory activity was taken to the next step of purification.

#### 2.8.2. Gel Filtration Chromatography

Peptide fraction with the highest activity of ACEinhibiting properties was subsequently separated on Sephadex G10. 2 mL of fraction was loaded on the column (1 × 120 cm) previously washed with distilled water that was used also as an eluent, with flow rate of 0.8 mL/min. One milliliter of each fraction was collected and the absorbance was monitored at 220 nm. Fractions with the highest absorbance were combined and ACE inhibitory activity was determined. Fractions with the highest ACE inhibitory activity were lyophilized and used in further analysis.

### 2.9. Kinetics of ACE Inhibition

Hippuryl-His-Leu solutions (1.0, 2.0, 3.0, 4.0, and 10.0 mM) were prepared and used to determine the Michaelis constant (*K*_*m*_), the inhibition constant (*K*_*i*_), and the maximum velocity (*V*_max⁡_) of ACE. The kinetic parameters were evaluated by Lineweaver-Burk's method. The reaction conditions were the same as ACE inhibitory activity assay. Briefly, 50 *μ*L of peptide sample (or 0.1 M borate buffer pH 8.3 for enzyme activity) was added to 50 *μ*L HHL solution (1.0, 2.0, 3.0, 4.0, or 10.0 mM) and 50 *μ*L of 3 mU/mL ACE (one unit of ACE activity was defined as an increase absorbance of 0.001 per minute at 390 nm). The reaction mixture was incubated at 37°C for 60 min. The reaction was terminated by adding 0.7 mL of the 0.1 M borate buffer with 0.2 M NaOH. The absorbance was measured at 390 nm.

### 2.10. Amino Acid Composition

The samples (50 *μ*g) were hydrolyzed in gas phase using 6 M HCl at 115°C for 24 h. The liberated amino acids were converted into phenylthiocarbamyl (PTC) derivatives and analyzed by high-pressure liquid chromatography (HPLC) on a PicoTag (3.9 × 150 mm) column (Waters, Milford, MA, USA) according to method of Boogers et al. [22].

### 2.11. Mass Spectrometry Analysis (ESI/MS-MS)

The molecular mass andpeptide sequencing were estimated by positive ion mode using electrospray ionisation-mass spectrometry. Lyophilized samples (0.1 mg) were dissolved in 0.3 mL of 50% acetonitrile containing 0.1% formic acid (v/v) and analysed using Applied Biosystems mass spectrometer, type Star XL MS-MS with nanoelectrospray ionization (nano-ESI). The analysis was carried out in duplicate in the positive ion mode under the following conditions: the potential of the cone (DP): 60 V, the voltage on the needle during sputtering (IS): 900–1500 V, and scan mode: TOF-MS (product ion). Ions were monitored in the field: 100–3500* m*/*z* (TOF-MS), 50–2500* m*/*z* (product ion), and collision energy (CE): 51.2 V. Acquired raw data were processed by Mascot Distiller followed by Mascot Search (Matrix Science, London, UK, onsite license) against NCBInr database (20120224). Search parameters for precursor and product ions mass tolerance were 40 ppm and 0.6 Da, respectively, enzyme specificity was semitrypsin, missed cleavage sites allowed were 0, and fixed modification of cysteine was by carbamidomethylation and variable modification of lysine carboxymethylation and methionine oxidation. Peptides with Mascot Score exceeding the threshold value corresponding to <5% false positive rate, calculated by Mascot procedure, and with the Mascot score above 30 were considered to be positively identified.

### 2.12. Statistical Analysis

Each treatment was conducted in triplicate and the results were presented as mean ± standard deviation. STATISTICA 7.0 was used for statistical analysis. Tukey's test was used to estimate significant differences among the mean values at the 5% probability level (α < 0.05).

## 3. Results and Discussion

Most studies of bioavailability of peptides released during protein hydrolysis process relate to changes caused by a single proteolytic enzyme or subsequent hydrolysis resulted in proteases of the digestive system, bypassing the step α-amylase hydrolysis. The α-amylase is not a proteolytic enzyme, but, in order to reflect the processes occurring in the gastrointestinal tract more accurately, it was also used in this study. The globulins hydrolysates obtained from pea were digested under simulated gastrointestinal digestion condition (α-amylase, pepsin, and pancreatin). The degree of hydrolysis was noted as follows: after pepsin, 14.42 ± 0.14%, and after pancreatin, 30.65 ± 0.33%. Similar results were obtained by Lo et al. [23] for soybean protein isolate; the value of DH after pepsin digestion was 11.0%. According to Barbana and Boye [12], DHs of proteins two varieties of chickpea (Xena kabuli and Myles desi) and the yellow pea var. Golden after simulating human gastrointestinal digestion were noted to be 34.41% ± 0.15, 40.78% ± 0.03, and 31.08% ± 0.05, respectively. The differences in results may be due to the fact that the preparation of samples, the method used for isolation of peptides, different protein fraction source, or varieties of pea could have an influence on the DHs value. It should be noted that the research about pea proteins hydrolysates obtained under gastrointestinal condition did not include stimulated saliva solution.

Potentially bioactive fragments of proteins with structural motifs corresponding to the bioactive peptides are inactive precursor protein in the sequence and only released by the action of proteolytic enzymes which may exhibit diverse biological activity [24, 25]. In this study, nonhydrolyzed protein showed no ACE inhibitory activity. Moreover, after* in vitro* digestion and absorption, the IC_50_ ACE peptides inhibitory value was noted to be 0.72 mg/mL, as enalapril with IC_50_ value of 7.46 mg/mL was used as a control. This result corresponds well with results obtained by Barbana and Boye [12], where IC_50_ values were determined for two varieties of chickpea and yellow pea for nonhydrolyzed proteins and after gastrointestinal digest. The results showed that only hydrolysates have ACE inhibitory activity and IC_50_ were noted for chickpeas to be 0.229 mg/mL and 0.14 mg/mL and for yellow pea 0.159 mg/mL [12]. Akillioğlu and Karakaya [13] reported that common bean and green lentils proteins hydrolyzed under 50 minutes of intestine digestion had ACE inhibitory activity with IC_50_ = 0.77 mg/mL and 0.26 mg/mL, respectively. As it was previously indicated, the legumes food proteins are a good source of ACE inhibitory peptides released after digestion and they must enter the circulatory system to have benefit to health.

**Figure 1:**
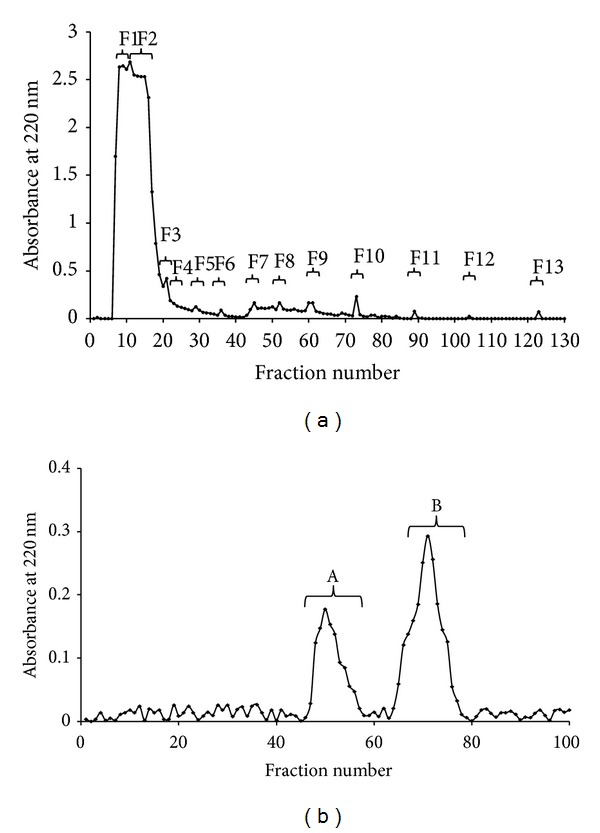
Purification of ACE inhibitory peptides from pea globulins hydrolysates separation on DEAE-cellulose (a) and F8 separation on Sephadex G10 (b).

For the purification of ACE inhibitory peptides, potentially bioavailable ion exchange chromatography with DEAE-cellulose and Sephadex G10 was used. As shown in Figure 1(a), the hydrolysates were fractionated into thirteen peptides fractions. For each fraction, peptides content and ACE inhibitory activity were determined (Table 1). Only five fractions showed ACE inhibitory activity. Although the highest peptides content (L-leucine as a standard) was noted in the first fraction (5.24 ± 0.42 mg/mL), the potentially highest ACE inhibitory peptide activity was determined for the eighth fraction (F8), where IC_50_ value was noted to be 0.0014 mg/mL. It should be noted that in the peptide content in most peptide fractions no significant difference was determined. F8 was not characterized by the highest peptides content; it suggests that the ACE inhibitory activity depends on the amino acids sequences. This fraction was separated using gel filtration on Sephadex G10 (Figure 1(b)). After this step, two peptides fractions were obtained but only the second fraction (B) exhibited potential hypertensive properties, IC_50_ = 0.073 mg/mL, and the fraction was used for further analysis. Higher IC_50_ value at this stage than after ion chromatography separation can be caused by synergistic action of peptides in the mixture.

**Figure 2:**
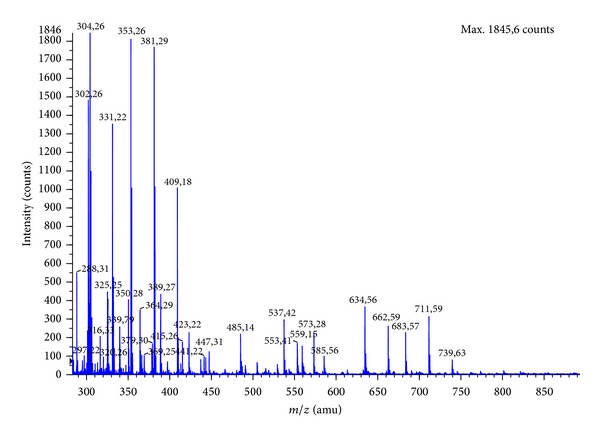
Nano-ESI-MS/MS spectrum of fraction B.

**Figure 3:**
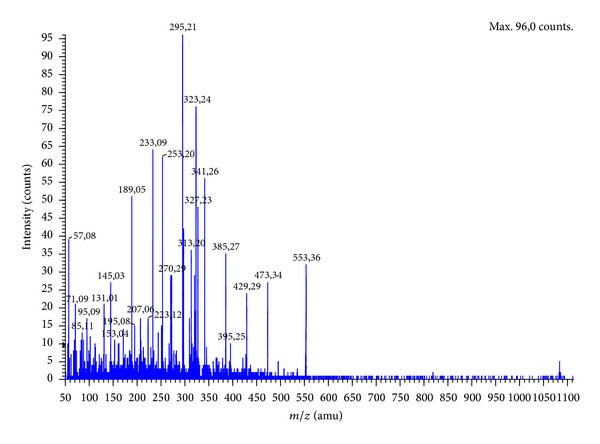
Representative fragmentation spectra of 553.41* m*/*z*.

An important step in the identification of peptides with high ACE inhibitory properties is determination of their amino acid composition. In this study, amino acid composition of peptide fractions with the highest ACE inhibition activity obtained after the separation on Sephadex G10 was determined by hydrolyzing them using 6 M HCl and then analyzed by HPLC (Table 2). Due to the fact that during hydrolysis process some amino acids as asparagine and glutamine are partially converted to aspartic acid and glutamic acid, respectively, the data for asparagine and/or aspartic acid were shown as Asx while those for glutamine and/or glutamic acid were reported as Glx. Hydrophilic amino acids were the main part in composition of fraction B. The highest content amino acids were Glx (20.78 ± 0.03%), alanine (16.69 ± 0.02%), glycine (11.14 ± 0.02%), serine (7.23 ± 0.01), Asx (10.41 ± 0.02), and arginine (7.47 ± 0.02). However, between Asx and argininine, no statistical significant difference was noted. On the other hand, threonine and phenylalanine were not detected. This corresponds well with results obtained by Wu and Ding [26]. Cited investigators also reported amino acid composition of potent ACE inhibitory peptides fraction obtained after separation of the hydrolysates on a cation exchange resin. The hydrophilic amino acids content was determined: arginine 19.67%, lysine 11.85%, and Glx 10.54% [26]. Important aspect in the explanation mechanism of ACE inhibition is to understand the construction of peptide inhibitors. The most common technique for the identification of active peptides is mass spectrometry.  Figure 2 illustrates the mass spectrum of fraction B. The analysis of ion sequencing was made using the Mascot database. In this study, the sequence of potential ACE inhibitory peptides was determined as follows: GGSGNY (553.41* m*/*z*), DLKLP (585.56* m*/*z*), GSSDNR (634.56* m*/*z*), MRDLK (662.59* m*/*z*), and HNTPSR (711.59* m*/*z*).  Figure 3 illustrates representative fragmentation spectra. The relationship between structure and activity of peptide inhibitors of ACE indicates that binding of the enzyme molecule is dependent on the type of amino acid C-terminal tripeptide sequence of the peptide. The strongest inhibitors of ACE contain hydrophobic (aromatic or branched side chains containing) amino acid residues at the C-terminus; the most preferred is proline [27]. In this study, one peptide containing C-terminal proline was identified as DLKLP. Some studies indicate that the occurrence of certain amino acids at the C-terminus or in a peptide does not necessary mean that the relationship had a strong hypotensive effect. Guang and Phillips [28] isolated a peptide with ACE inhibitory activity from hazelnut with sequence KAFR. Another example of a peptide without proline at the C-terminus is pentapeptide with sequence LVQGS isolated from the extract of fermented soybean seeds [29]. Moreover, Li et al. isolated from alcalase hydrolysates of mung bean proteins ACE inhibitory peptides with sequence KDRL, VTPALR, and KLPAGTLF [30].

**Figure 4:**
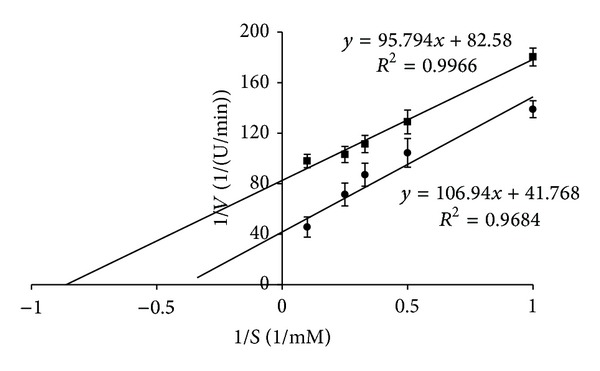
The Lineweaver-Burk plot for the inhibition of ACE (●—without inhibitor; ■—with fraction B of* in vitro* digested pea globulins).

Lineweaver-Burk plots of the ACE inhibition pattern of the fraction B were shown in Figure 4. The data (Table 3) revealed that *K*_*i*_ of the peptide fraction resulted in reduction of the value of *K*_*m*_ and the maximum rate of enzymatic reaction (*V*_max⁡_); therefore, this fraction was uncompetitive inhibitor of ACE. This means that inhibitor binds only to the enzyme-substrate complex (E-S) and not to the free enzyme. Although ACE inhibitory peptides have been reported as competitive inhibitors [11, 25], most ACE inhibitory peptides belong to uncompetitive inhibitors. According to Pedroche et al. [31], two ACE inhibitory peptides fractions from chickpea hydrolysates were uncompetitive inhibitors. Furthermore, alcalase hydrolysates of soybean proteins were also a source of peptides that inhibit ACE activity as uncompetitive inhibitors [26, 32].

## 4. Conclusions

As a result of the conducted research, it can be stated that the seeds of pea are the source of precursor protein, which releases the ACE inhibitory peptides as a result of enzymatic hydrolysis. In this study, ACE inhibitory peptides were identified by ESI-MS/MS. The sequences of novel ACE inhibitory peptides were GGSGNY, DLKLP, GSSDNR, MRDLK, and HNTPSR. Based on Lineweaver-Burk plots for the fraction B, the kinetic parameters as *K*_*m*_, *V*_max⁡_, and *K*_*i*_ and mode of inhibition were determined. This fraction belongs to uncompetitive inhibitor of ACE activity.

## Figures and Tables

**Table 1:** Peptides concentration and IC_50_ value of peptides inhibitory activity fractions obtained after separation on DEAE-cellulose.

Fraction number	Peptides content (mg/mL)	IC_50_ (mg peptide/mL)
F1	5.24 ± 0.42^a^	0.573 ± 0.017^A^
F2	0.14 ± 0.01^b^	ND
F3	0.006 ± 0.0006^b^	0.0045 ± 0.0009^B^
F4	0.009 ± 0.0003^b^	ND
F5	0.014 ± 0.0006^b^	ND
F6	0.018 ± 0.0005^b^	ND
F7	0.015 ± 0.0014^b^	0.0026 ± 0.0004^C^
F8	0.024 ± 0.008^b^	0.0014 ± 0.0003^D^
F9	0.022 ± 0.004^b^	ND
F10	0.024 ± 0.005^b^	ND
F11	0.027 ± 0.007^b^	ND
F12	0.024 ± 0.0007^b^	ND
F13	0.022 ± 0.0013^b^	0.0018 ± 0.0003^E^

ND: not noted.

All values are mean ± standard deviation for triplicate experiments.

Values with different letters superscripts are significantly different at *α* < 0.05.

**Table 2:** Amino acid composition (%) of fraction B.

Amino acid	% of amino acid composition
Asx∗	10.41 ± 0.02^e^
Glx∗	20.78 ± 0.03^g^
S	7.23 ± 0.01^d,e^
G	11.14 ± 0.02^e^
H	1.02 ± 0.004^a,b^
R	7.47 ± 0.02^d,e^
T	ND
A	16.69 ± 0.02^f^
P	5.18 ± 0.01^c,d^
Y	2.08 ± 0.002^a,b,c^
V	4.25 ± 0.006^b,c,d^
M	0.32 ± 0.002^a^
I	3.93 ± 0.005^a,b,c,d^
L	5.18 ± 0.009^c,d^
F	ND
K	4.32 ± 0.008^b,c,d^

∗Asx: D + N; Glx: E + Q.

ND: not detected values.

All values are mean ± standard deviation for triplicate experiments.

Values with different letters superscripts are significantly different at *α* < 0.05.

**Table 3:** ACE inhibitory constants for fraction B.

	Control	Fraction B
IC_50_ (mg/mL)	—	0.073 ± 0.002
*K*_*i*_ (mg/mL)	—	0.039 ± 0.003
*K*_*m*_ (mM)	2.56 ± 0.32^a^	1.16 ± 0.11^b^
*V*_max⁡_ (U/min)	0.024 ± 0.004^A^	0.012 ± 0.002^B^

All values are mean ± standard deviation for triplicate experiments.

Values with different letters superscripts are significantly different at *α* < 0.05.
